# Perceptions of Patients and Physicians Regarding Audio-Only Gynecology Virtual Clinics: The Experience of a Tertiary Hospital in Jeddah, Saudi Arabia

**DOI:** 10.7759/cureus.113399

**Published:** 2026-07-26

**Authors:** Asma M Khalil, Shahad S Alharazi, Farah H Bakhamees

**Affiliations:** 1 Department of Obstetrics and Gynecology, King Abdulaziz Medical City, Jeddah, SAU; 2 King Abdullah International Medical Research Center, Ministry of National Guard Health Affairs, Jeddah, SAU; 3 Department of Family Medicine, Prince Mohammed Bin Abdulaziz Hospital, Al-Madinah, SAU

**Keywords:** gynecology, patient, perception, physician, saudi arabia, telehealth, telemedicine

## Abstract

Background: Since the outbreak of coronavirus disease 2019 (COVID-19), virtual clinics served as the cornerstone modality of telehealth in many health institutions. This study aimed to examine patients' and physicians' perceptions of the integration of audio-only gynecology virtual clinics.

Methods: A cross-sectional study was conducted in a tertiary hospital in Jeddah, Saudi Arabia. A convenience sampling technique was followed for physicians and patients. The questionnaires utilized were validated.

Results: During March 2021-March 2022, a total of 220 subjects participated in the study. Physicians accounted for 24.5% (n = 54), while patients accounted for 75.5% (n = 166). Overall, only 55.5% of the physicians agreed virtual clinics should continue after the pandemic, while 84.6% and 76% of the patients reported satisfaction and the wish to use virtual clinics again, respectively. Physicians who seldom found physical examination to be indicated, who seldom requested patients to attend physically to the clinic, and who had a positive outlook on virtual clinics were more likely to desire continuing virtual clinics after the pandemic (p < 0.001). Patients who had positive outlooks regarding virtual clinics were shown to be more satisfied with virtual clinics (p < 0.001).

Conclusions: Audio-only gynecology virtual clinics are accepted by both physicians and patients with higher satisfaction among patients. Physicians' positive outlook yet reluctance to continue virtual clinics suggests the need to modify the conduction of virtual clinics and necessitates larger-scale prospective studies on this topic.

## Introduction

Since the first reports of coronavirus disease 2019 (COVID-19) in late 2019, caused by severe acute respiratory syndrome coronavirus 2 (SARS-CoV-2), in Wuhan, China, the disease has rapidly evolved into a global pandemic and a major public health challenge worldwide [1]. COVID-19 has resulted in hundreds of millions of confirmed cases and millions of deaths globally, placing an unprecedented burden on healthcare systems and societies [1,2]. The casualties of the pandemic also extended beyond confirmed COVID-19 cases, deaths, and catastrophic economic and social impacts and reached healthcare systems [3].

Due to the highly infective nature of COVID-19, one of the most prominent changes that were mandated in healthcare systems is the rapid integration of "telehealth" [4]. Telehealth has been described in the literature as a "technology-enhanced healthcare framework," which encompasses distant monitoring of patients, virtual clinics and consultations, and personal electronic health records accessible by patients through their mobile phones, computers, and other digital devices [4].

Virtual clinics have been a cornerstone modality of telehealth in many health institutions during the COVID-19 outbreak [5,6]. Nonetheless, patients' and physicians' satisfaction with the quick transition from in-person regular clinics to virtual clinics have been noted in literature across a number of different medical and surgical specialties, including gastroenterology [6], endocrinology [7], urology [8], emergency medicine [9], rheumatology [10], pediatrics [11], and obstetrics and gynecology [4,5,12-17].

A study conducted in urology revealed that tele-consultation was perceived to have been a good experience by 80% of the physicians and 84% of the patients. Different specialties perceived the impact and level of challenge in regard to the patient-physician relationship, delivery manner, and diagnostic process differently [17]. Along with surgeons, obstetricians and gynecologists felt the least challenged [17]. Telehealth found its firm footing in gynecology through its many applications such as preconception counselling, infertility workup, mental health, preventive care, family and contraception planning, teleradiology, telesurgery, cervical cancer screening and colposcopy, and, particularly, well-woman care [14]. Telehealth integration into gynecology was noted to be effective not only for the continuation of both injectable and oral contraception but also to increase oral contraception rates at the six-month point [18]. It is no surprise that telehealth integration into the field of obstetrics and gynecology was shown to be highly accepted and valued by both physicians and patients [7]. In fact, compared to traditional care, patients who utilized antenatal virtual clinics were shown to report higher satisfaction rates (79% versus 94%; p < 0.01), lower pregnancy-related stress, increased nursing time (108 minutes versus 171 minutes), and similar perceived quality of care and clinical outcomes [12].

To the researchers' knowledge, only one telemedicine-focused study was conducted in Saudi Arabia in which the positive experience and high satisfaction rates of healthcare providers and patients in diabetes virtual clinics were noted [7]. However, no study was conducted in Saudi Arabia that focused on physicians' and patients' perceptions and satisfaction with gynecological virtual clinics. Therefore, this study aimed to examine patients' and physicians' perceptions of telemedicine by determining each group's perception and satisfaction level towards the recent integration of audio-only gynecology virtual clinics in a tertiary healthcare hospital in Jeddah, Saudi Arabia. The primary outcome was overall satisfaction with audio-only gynecology virtual clinics. Secondary outcomes included willingness to continue using virtual clinic services, perceptions of healthcare accessibility and quality of care, and physicians' and patients' preferences regarding the future use of audio-only virtual clinics.

This article was previously released as a preprint in November 2024.

## Materials and methods

This is a cross-sectional study in which a non-probability convenience sampling technique was followed for the quantitative data collection. After obtaining institutional review board approval and the participants' approval, data was collected during a one-year period from March 2021 to March 2022 at King Abdulaziz Medical City in Jeddah, Saudi Arabia. The study was conducted in accordance with the Declaration of Helsinki and approved by the Institutional Review Board of King Abdullah International Medical Research Center (approval number: NRJ21J/010/01; date of approval: 11/02/2021). All participants were informed about the purpose of the study, and informed consent was obtained prior to participation. Participation was voluntary, and participants were informed of their right to withdraw at any time without consequences. To ensure confidentiality, all collected data were anonymized and stored securely, and no personally identifiable information was included in the analysis or reporting of the results.

Audio-only gynecology virtual clinics were conducted through scheduled telephone consultations by obstetrics and gynecology physicians. Clinical encounters were documented in the hospital's electronic medical record system in accordance with standard outpatient documentation practices. Patients requiring physical examination, diagnostic procedures, urgent assessment, or further management beyond the scope of a telephone consultation were referred for in-person clinic evaluation. Follow-up appointments were arranged according to the patient's clinical condition and physician judgment. Virtual consultations were conducted according to institutional policies and routine clinical practice during the COVID-19 pandemic.

The study's target population was divided into two groups: the patient group and the physician group. The patient group included all female patients who virtually visited the gynecology clinic of the tertiary hospital during the COVID-19 pandemic. Walk-in female patients who had no gynecology virtual clinic experience and who only physically attended the obstetrics and gynecology clinic without a previous virtual clinic appointment or visit were excluded. A sample size calculation was performed assuming a 95% confidence level, a 5% margin of error, and a 50% response distribution, and the minimum required patient sample size was estimated to be 160 participants. As for the physician group, it included obstetrics and gynecology residents, specialists, fellows, and consultants of all subspecialties who have attended to the virtual clinics. Interns and rotating residents were excluded. A convenience sampling approach was followed, and all eligible physicians available during the study period were invited to participate.

Two forms of the questionnaire were utilized, Version A for the patients and Version B for the physicians. Version A (patients' questionnaire) incorporated two validated questionnaires, the Short Assessment of Patient Satisfaction (SAPS) and the Antenatal and Neonatal Guidelines, Education, and Learning Systems Patient Satisfaction with Telehealth Questionnaire (ANGELS PSTQ) [19,20]. Both of the questionnaires were translated into Arabic prior to administration to facilitate participant understanding. However, no formal back-translation procedure, pilot testing, cultural validation, or assessment of internal consistency was performed before its use in the study population. Version B (physicians' questionnaire) consisted of one validated questionnaire, the Antenatal and Neonatal Guidelines, Education, and Learning Systems Provider Satisfaction with Telehealth Questionnaire (ANGELS PrSTQ), which was delivered in its original format in English [20].

The patients' version of the questionnaire (Version A) was either self-administered or interviewer-administered. Physical copies of the questionnaire were made available at the gynecology clinics and were provided by the nurses to patients who had previous virtual visits to the gynecology clinic. Moreover, patients with previous experience with gynecology virtual clinics were contacted through phone calls. A structured interview approach based on the question outline of the questionnaire was followed, and the responses were administered by the interviewer. On the other hand, the physicians' version of the questionnaire (Version B) was self-administered. An electronic version of the questionnaire was supplied to all physicians who attended to gynecology clinics.

Statistical analysis

Data was extracted from the physical and electronic data collection sheets and then coded and refined into two separate Microsoft Excel sheets (Microsoft Corp., Redmond, WA, USA) (one sheet for the physicians' responses and another sheet for the patients' responses). Data was first cleaned and then exported into IBM SPSS Statistics for Windows, V. 23.0 (IBM Corp., Armonk, NY, USA), for statistical analysis. Categorical data was described by means of percentages and pie charts. As for inferential statistics, variables were compared using Fisher's exact test. A p-value (p) of less than 0.05 was considered statistically significant.

## Results

From March 2021 to March 2022, a total of 220 study subjects participated in the study. Physicians accounted for 24.5% of the population (n = 54), while patients accounted for 75.5% (n = 166). The demographic data of the two populations is summarized in Table 1.

**Table 1:** Socio-demographic profile of the respondents (n = 220) Denominators may vary across variables because of missing responses. Percentages were calculated using the number of available responses for each variable.

			Frequency (n)	Percentage (%)
Physicians* *(n = 54; 24.5%)	Gender	Female	40	74.1
		Male	14	25.9
	Age	25-34 years	35	64.8
		35-44 years	9	16.7
		45-54 years	6	11.1
		More than 54 years	4	7.5
	Position	Residents	33	61.1
		Registrars and consultants	21	38.9
	Years of experience	Less than 2 years	8	14.8
		2-5 years	21	38.9
		6-10 years	9	16.7
		More than 10 years	16	29.6
Patients* *(n = 166; 75.5%)	Age	Under 20 years	5	3
		20-29 years	46	27.9
		30-39 years	53	32.1
		40-49 years	38	23
		More than 50 years	23	13.9
	Educational level	Less than high school	34	20.6
		High school degree	43	26.1
		Diploma	10	6.1
		Bachelor degree	71	43
		Postgraduate studies	7	4.2
	Residence	Jeddah	113	68.1
		Outside Jeddah	53	31.9
	Transportation mode	On foot	1	0.6
		By car (inside Jeddah)	115	69.3
		By car (outside Jeddah)	48	28.9
		Air transportation	2	1.2
	Missed days of work	0 days	91	70.5
		1 day	31	24
		2-3 days	4	3.1
		4 days and more	3	2.3
	Visit type	First visit	23	13.9
		Follow-up	142	86.1
	Number of virtual visits received	1 visit	54	34
		2-5 visits	89	56
		6-10 visits	13	8.2
		More than 10 visits	3	1.9

**Figure 1:**
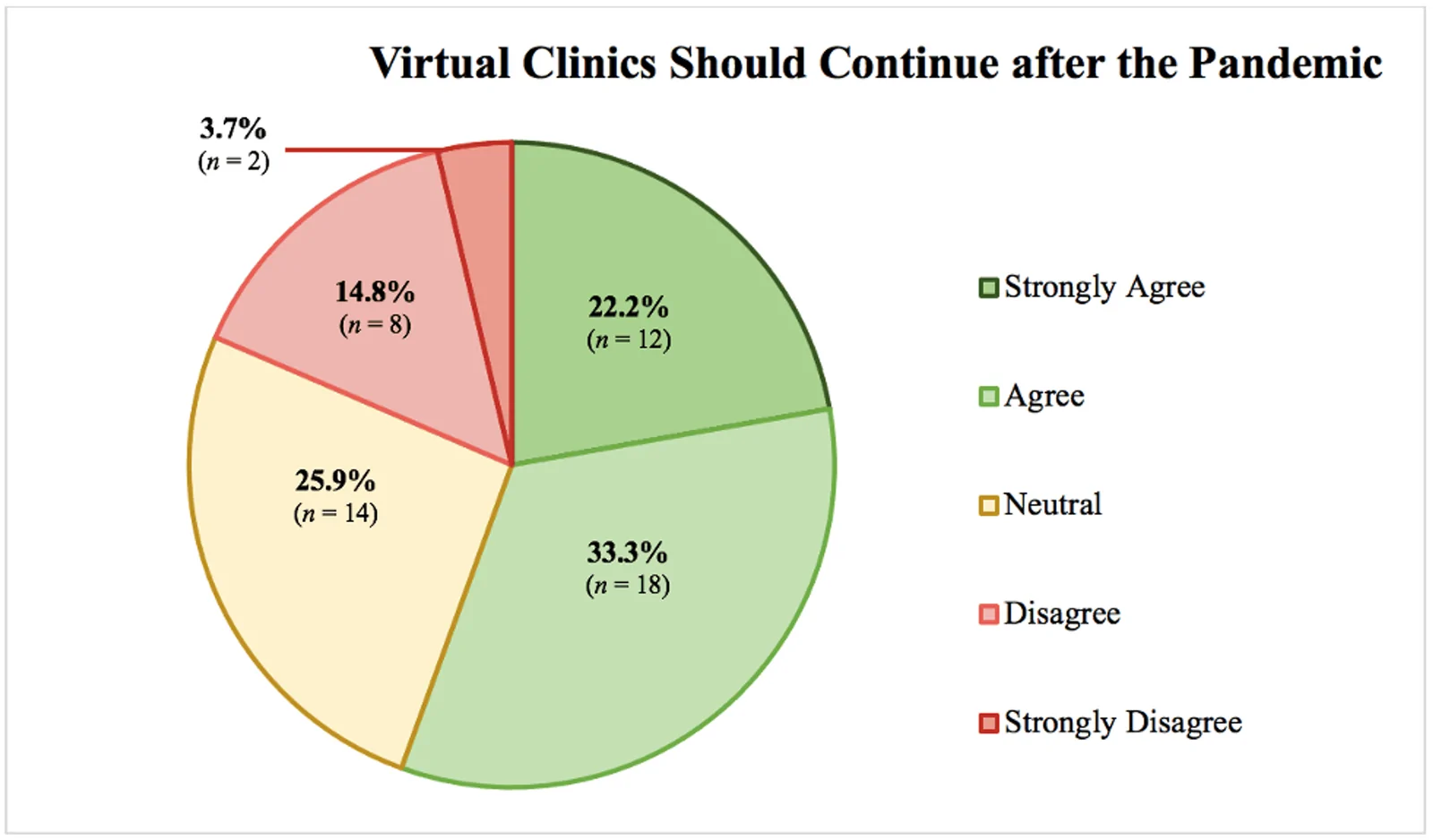
Physicians' preference in regard to the continuation of audio-only gynecology virtual clinics after the pandemic (n = 54)

Physicians' experiences and perceptions of audio-only virtual gynecology clinics are shown in Table 2. More than half of the physicians (61.1%) reported that they were able to reach the patient from the first call about half the time only, while only 37% expressed that they were usually able to speak to the patient. Almost half of the physicians (48.1%) found that physical examination was indicated about half the time and that they have seldom asked the patient to attend physically to the clinic. Overall, 55.5% of the physicians either agreed or strongly agreed that virtual clinics should continue after the pandemic, while only 18.5% of them either disagreed or strongly disagreed with the continuation, as shown in Figure 1. Neither gender nor age showed statistical significance; however, registrars and consultants were more likely to agree that virtual clinics are more convenient to see a larger number of patients in an optimal way, with a statistical significance of p = 0.029. Moreover, the association between years of experience and ability to speak directly to the patient was statistically significant (p = 0.013), with those with two to five years of experience being more likely to only be able to speak directly to the patient half of the time. Physicians who seldom found physical examination to be indicated or requested patients to attend physically to the clinic were more likely to desire continuing virtual clinics after the pandemic, with a strong statistical significance of p < 0.001. Furthermore, physicians who believed that virtual clinics improve healthcare access to patients, are an acceptable way to provide healthcare services, are a convenient method of seeing a larger number of patients in an optimal way, and are an adequate replacement when in-patient visits are difficult were all more likely to desire continuing virtual clinics after the pandemic, with a strong statistical significance of p < 0.001.

**Table 2:** Physicians' experiences and perceptions of audio-only virtual gynecology clinics (n = 54)

	Always	Usually	About half the time	Seldom	Never	P-value
	n (%)	n (%)	n (%)	n (%)	n (%)	
Ability to reach the patient immediately from the first call	0 (0)	11 (20.4)	33 (61.1)	0 (0)	0 (0)	0.050
Ability to speak directly to the patient	5 (9.3)	20 (37)	16 (29.6)	13 (24.1)	0 (0)	0.270
Indication of physical examination	0 (0)	3 (5.6)	26 (48.1)	24 (44.4)	1 (1.9)	<0.001
Asking the patient to attend physically to the clinic	1 (1.9)	2 (3.7)	25 (46.3)	26 (48.1)	0 (0)	<0.001
	Strongly agree	Agree	Neutral	Disagree	Strongly disagree	P-value
	n (%)	n (%)	n (%)	n (%)	n (%)	
Virtual clinics improved healthcare access for patients' needs	12 (22.2)	15 (27.8)	21 (38.9)	5 (9.3)	1 (1.9)	<0.001
Virtual clinics are an acceptable way to provide healthcare services	15 (27.8)	19 (35.2)	17 (31.5)	3 (5.6)	0 (0)	<0.001
Virtual clinics are able to provide optimal care to patients	6 (11.1)	18 (33.3)	19 (35.2)	11 (20.4)	0 (0)	0.066
Virtual clinics are more convenient to see a larger number of patients in an optimal way	10 (18.5)	11 (20.4)	16 (29.6)	16 (29.6)	1 (1.9)	<0.001
Virtual clinics are an adequate replacement when in-patient visits are difficult	19 (35.2)	19 (35.2)	8 (14.8)	7 (13)	1 (1.9)	<0.001

**Figure 2:**
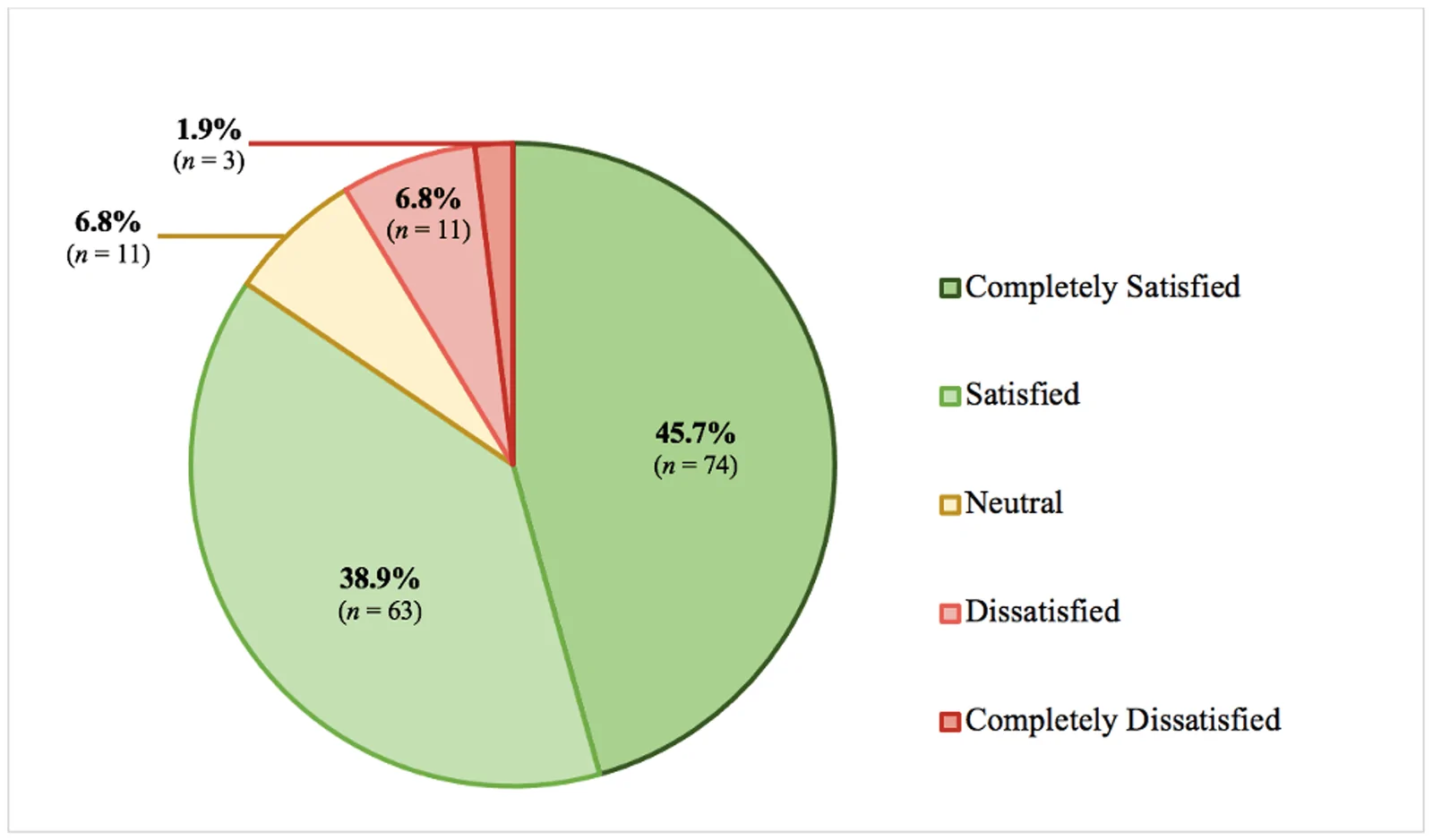
Patients' overall satisfaction with audio-only gynecology virtual clinics (n = 162)

The overall percentage of patients who have reported overall complete satisfaction with the gynecology virtual clinics was 84.6%, as shown in Figure 2. Age, educational level, residence, transportation mode, missed days of work, visit type, and number of virtual visits all failed to show any statistical significance to satisfaction level. Patients' experiences, perceptions, and satisfaction with audio-only gynecology clinics are outlined in Table 3. Patients who reported having been called at the registered appointment time were more likely to report higher satisfaction, with a statistical significance of p = 0.013. The availability of gynecology virtual clinics within the Saudi healthcare system appears to be an important factor influencing patient perceptions. Participants who viewed virtual clinics as an acceptable method of receiving healthcare services demonstrated significantly higher satisfaction levels (p < 0.001). Moreover, patients who felt that they can easily talk to their physicians in virtual clinics, that physicians were keen to hear all concerns during virtual clinics, that all their questions were answered, and that physicians clearly explained all required examinations and follow-up plans during virtual clinics reported statistically significant satisfaction rates (p < 0.001). Patients who found the time lengths of audio-only virtual visits suitable and who were requested to come personally to the healthcare center for further examination also reported statistically significant satisfaction rates (p = 0.001 and p < 0.001, respectively). Moreover, those who found that virtual clinics saved transportation and waiting times and made it easy to attend all appointments, who had similar levels of satisfaction when compared to in-hospital visits, who were happy to use virtual clinics again for the same complaint, and who were satisfied with physicians' explanation of results and treatment plans, discussion of treatment options, and effect of the treatment and care provided during the virtual clinic all reported statistically significant satisfaction levels when compared to their counterparts (p < 0.001). Lastly, patients' perception of physicians' respect during virtual clinics was shown to be statistically correlated with significant satisfaction levels (p = 0.043).

**Table 3:** Patients' experiences, perceptions, and satisfaction with audio-only virtual gynecology clinics (n = 166)

	Yes			No		P-value
	n (%)			n (%)		
Receipt of a text directing the patient not to come to the healthcare center	130 (80.7)			31 (19.3)		0.904
Physician calling at the registered appointment time	142 (86.6)			22 (13.4)		0.013
Physician introducing himself or herself	151 (94.4)			9 (5.6)		0.871
Occurrence of any technical issue	23 (14.1)			140 (85.9)		0.582
	Strongly agree	Agree	Neutral	Disagree	Strongly disagree	P-value
	n (%)	n (%)	n (%)	n (%)	n (%)	
The availability of virtual clinics in the Saudi healthcare system is important for gynecology patients	65 (39.6)	70 (42.7)	14 (8.5)	12 (7.3)	3 (1.8)	<0.001
Virtual clinics are an acceptable way to receive healthcare services	60 (36.4)	82 (49.7)	15 (9.1)	7 (4.2)	1 (0.6)	<0.001
I can talk to physicians easily through the virtual clinic	64 (39)	75 (45.7)	15 (9.1)	8 (4.9)	2 (1.2)	<0.001
Physicians were keen to hear all concerns during virtual clinics	78 (47.6)	71 (43.3)	9 (5.5)	5 (3)	1 (0.6)	<0.001
Physicians answered all questions during virtual clinics	75 (45.7)	76 (46.3)	10 (6.1)	2 (1.2)	1 (0.6)	<0.001
Physicians clearly explained the required medical examinations and follow-up plan during virtual clinics	68 (42)	70 (43.2)	19 (11.7)	4 (2.5)	1 (0.6)	<0.001
The time spent with the physician on the phone call was too short	14 (9)	37 (23.9)	21 (13.5)	58 (37.4)	25 (16.1)	0.001
Physicians asked to personally come to the clinic for further examination	50 (31.3)	69 (43.1)	6 (3.8)	25 (15.6)	10 (6.3)	<0.001
Virtual clinics saved the travel time required to get to the hospital	61 (39.4)	51 (32.9)	23 (14.8)	14 (9)	6 (3.9)	<0.001
Virtual clinics saved the waiting time spent in clinics	75 (47.2)	62 (39)	12 (7.5)	9 (5.7)	1 (0.6)	<0.001
Virtual clinics made it easy to attend all appointments	75 (46.9)	62 (38.8)	12 (7.5)	8 (5)	3 (1.9)	<0.001
The level of satisfaction with virtual clinics is similar to in-hospital visits and seeing physicians in person	57 (35)	60 (36.8)	18 (11)	17 (10.4)	11 (6.7)	<0.001
I am happy to use the virtual clinic again in the event that medical care is needed for the same complaint	62 (38)	62 (38)	21 (12.9)	14 (8.6)	4 (2.5)	<0.001
	Completely satisfied	Satisfied	Neutral	Dissatisfied	Completely dissatisfied	P-value
	n (%)	n (%)	n (%)	n (%)	n (%)	
Satisfaction with the physicians' explanation of the results of the treatment plan and care provided during the virtual clinic	79 (48.8)	63 (38.9)	11 (6.8)	7 (4.3)	2 (1.2)	<0.001
Satisfaction with the discussion of the treatment options provided by the physician during the virtual clinic	75 (46.3)	62 (38.3)	15 (9.3)	9 (5.6)	1 (0.6)	<0.001
Satisfaction with the effect of the treatment and care provided during the virtual clinic	70 (43.8)	63 (39.4)	21 (13.1)	5 (3.1)	1 (0.6)	<0.001

## Discussion

Telemedicine was found to achieve the goals of improving care experience, engagement, and satisfaction, reducing costs and time spent in transportation, and providing health outcomes comparable to traditional methods without compromising the quality of care nor the patient-physician relationship [4,6]. Overall, physicians' desire to continue pursuing, practicing, and offering telemedicine services, such as virtual clinics, has been noted in the literature [16]. The desire to continue telemedicine after the resolution of the pandemic was perceived to be associated with higher satisfaction rates [6,16]. Therefore, it is no surprise that telehealth integration into the field of obstetrics and gynecology was shown to be highly accepted and valued by both physicians and patients [7].

In this study, more than half of the physicians (61.1%) reported that they were able to reach the patient from the first call about half the time only, while only 37% expressed that they were usually able to speak directly to the patient. This barrier is probably due to the conservative nature of the Saudi community, in which the spouse's phone number is in many times the number registered and contacted in many instances. The 38.9% of physicians who expressed not being able to reach patients from the first call might be explained by the unavailability of many patients in the morning hours due to being asleep, despite personally reserving morning appointments. This leads to many calls of the virtual clinic being unanswered. As for physicians, the inability to perform physical examination was the biggest telemedicine drawback reported in multiple studies [10,21]. The inability to access patient's medical records and to perform laboratory tests were also some of the biggest barriers reported in other studies [11,21]. Compared to previous studies and unlike our study, physicians reported that the inability to perform physical examination, access patient's medical records, and perform laboratory tests were the biggest telemedicine drawback reported in multiple studies [10,11,21]. Interestingly, female physicians felt more challenged in regard to their clinical skills when compared to male physicians (71% versus 51%; p = 0.002), in a previous study [22]. However, in our current study, no statistically significant association was noted between gender and any of the other variables.

In this current study, almost half of the physicians (48.1%) found that physical examination was indicated about half the time and that they have seldom asked the patient to attend physically to the clinic. Despite this noted drawback, these same physicians were noted to be more supportive of the continuation of virtual clinics. This might be explained by an outlook of "benefits outweigh the risks," due to the positive beliefs the majority seemed to hold, such as that virtual clinics improve healthcare access to patients, are an acceptable way to provide healthcare services, are a convenient method of seeing a larger number of patients in an optimal way, and are an adequate replacement when in-patient visits are difficult. Such positive attitudes toward virtual clinics were previously reported in the literature, where physicians also believed that telemedicine increased services' accessibility, improved healthcare quality, and enhanced ease of use for both physicians and patients [10,16].

However, despite the many benefits that physicians perceived in the use of telehealth in this current study, only about half of the physicians (55.5%) desired the continuation of virtual visits. This discrepancy is understandable and explainable as previously noted with patients of a previous study, as the integration of telemedicine during the pandemic aims to not fully replace in-person encounters but to simply integrate screening, monitoring, and examination into fewer in-person visits to reduce the risk of COVID-19 exposure to both the patient and physician [13]. However, in the current study, virtual clinics replaced in-person visits as a whole, except for the cases that required in-person examination. Therefore, the high proportion of physicians who perceived virtual clinics as beneficial, compared with the lower proportion who supported their long-term continuation, may be explained by the manner in which telehealth was implemented at the tertiary hospital and the extensive reliance on virtual clinics during the COVID-19 pandemic to reduce the risk of infection. It might be hypothesized that virtual clinics can therefore be better implemented after the pandemic by the incomplete reliance on their conduction, in order to continue reaping the many benefits that physicians agreed upon and to overcome the main reason as to why only half of the physicians wish virtual clinics to continue. Modifications of the conduction of virtual clinics might increase the satisfaction rates to the ones reported in the Republic of Turkey, where almost all (99%) physicians were satisfied with telemedicine applications during the pandemic.

In this current study, the overall percentage of patients who reported overall satisfaction and complete satisfaction with the gynecology virtual clinics was 84.6%, which is comparable to a study conducted in the Republic of Turkey, a country within the Middle East, in which 87% of their patients reported satisfaction [11]. Unlike another study conducted in Karachi, the capital of Pakistan and a developing country like Saudi Arabia, which showed that the biggest barriers found in the integration of telemedicine were poverty and lack of education [22], educational level failed to show any statistically significant correlation to satisfaction level in our current study. As for the difference in the level of satisfaction across age groups toward virtual clinics, a recent study revealed that older patients have become more accepting and satisfied, akin to all other age groups nowadays when compared to the results of studies conducted at the start of the pandemic and early integration of telemedicine [6]. This correlates well with our study, as all age groups showed acceptance and satisfaction.

Similar to a study conducted in the emergency department that revealed patients' satisfaction remained similar in in-person and virtual visits, 71.8% of the patients in our study reported that the level of satisfaction with virtual clinics is similar to in-hospital visits and seeing physicians in person. Moreover, in the current study, most of the patients (90.9%) felt that their concerns were heard, and 76% were happy to use virtual clinics again. Similar to our study outcomes, another study published reported that 80% of the patients felt that their concerns were heard and addressed by their provider and wished to continue incorporating virtual clinics into their future care [6]. To align with our study, almost all (99%) of the patients who participated in another study focusing on audio-only prenatal virtual clinics reported having had their needs assured [5]. Patients in the aforementioned study were also noted to attend their prenatal virtual appointments more when compared to in-person prenatal appointments (88% versus 82%; p < 0.001). This is analogous to what 85.7% of our patients reported when asked if virtual clinics made attending all appointments easier. Patients' most significant reported virtual clinic advantages in the literature were convenience, saving transportation time, and reduced costs associated with travel from remote areas, missed occupational commitments, and childcare arrangements [6,11]. Compared to our study, the highest agreed-upon benefit of virtual clinics is the saved time usually spent in clinics' waiting rooms, followed by the ability to attend all appointments, at 86.2% and 85.7%, respectively.

As for the strengths and limitations of this study, several limitations should be acknowledged. First, the cross-sectional design precludes the assessment of causal relationships. Second, the relatively small patient sample size may limit the generalizability of the findings. Third, the use of self-reported data may have introduced recall bias, as participants may not have accurately remembered their experiences with virtual clinics. Additionally, because some questionnaires were administered by interviewers over the phone, interviewer bias may have influenced participants' responses. Non-response bias cannot be excluded, as individuals who declined participation may have differed from those who completed the survey. Furthermore, the use of convenience sampling may have introduced selection bias, potentially affecting the representativeness of the study population. In addition, the statistical analysis was primarily based on univariate comparisons and did not include multivariable analyses to identify independent predictors of satisfaction and acceptance of telemedicine. Therefore, potential confounding factors such as age, educational level, number of visits, physician experience, visit type (first versus follow-up), and place of residence were not adjusted for. Future studies with larger sample sizes are warranted to perform multivariable analyses and better elucidate the factors influencing perceptions of telemedicine services. Lastly, although the patients' questionnaire was based on previously validated instruments and translated into Arabic, no formal back-translation, cultural validation, pilot testing, or reliability assessment was conducted in the local study population. Therefore, the validity and reliability of the translated questionnaire within the Saudi Arabian context could not be formally established. Despite these limitations, this study is the first, to our knowledge, to investigate both physicians' and patients' perceptions of gynecology virtual clinics in Saudi Arabia. Moreover, the use of both telephone interviews and physical questionnaires enabled the researchers to reach a broader segment of the target population.

## Conclusions

Audio-only gynecology virtual clinics are generally perceived favorably, particularly by patients, although physicians demonstrate more cautious support regarding long-term continuation. Moreover, patients' satisfaction is higher than physicians', with saving waiting time and the ability of attending all appointments being the biggest benefits reported. Physicians' positive outlook on virtual clinics but significant reluctance to continue using them suggests the need to modify the conduction of virtual clinics and necessitates large-scale prospective studies.
